# The Number of Monthly Night Shift Days and Depression Were Associated with an Increased Risk of Excessive Daytime Sleepiness in Emergency Physicians in South Korea

**DOI:** 10.3390/bs12080279

**Published:** 2022-08-11

**Authors:** Song Yi Park, Hyung Min Lee, Jiyoung Kim

**Affiliations:** 1 Department of Emergency Medicine, College of Medicine, Dong-A University, Busan 48114, Korea; 2 Department of Emergency Medicine, Hallym University Sacred Heart Hospital, Anyang 14068, Korea; 3 Department of Neurology and Sleep Disorder Center, Bio Medical Research Institute, Pusan National University Hospital, School of Medicine, Pusan National University, Busan 50612, Korea

**Keywords:** prevalence, emergency medicine, physicians, disorders of excessive somnolence, republic of Korea

## Abstract

This study aimed to report the prevalence and identify the factors associated with excessive daytime sleepiness (EDS) among emergency physicians in South Korea. We analyzed the Korean Emergency Physicians Survey data from 15 January to 26 February 2021. EDS was evaluated using the Epworth sleepiness scale, and a score of 11 or more indicated the presence of EDS. We conducted univariable and multivariable logistic regression analyses to verify the associated factors. A total of 1307 participants responded to the survey, and the response rate was 61.3%. Nine hundred fifty-four participants were included in the study. Two hundred ninety-three participants were classified as the EDS group, and six hundred sixty-one were classified as the non-EDS group. The prevalence of EDS was 30.7% (95% confidence interval (CI), 27.8–33.6%). Monthly night-shift days (odds ratio (OR) 1.106, 95% CI 1.028–1.191) and depression (OR 2.635, 95% CI 1.799–3.861) were significantly associated with an increased risk of EDS, and fair sleep quality (OR 0.560, 95% CI 0.318–0.985) was associated with a decreased risk of EDS. Almost one in three emergency physicians in South Korea suffer from daytime sleepiness. The number of monthly night-shift days and depression were associated with an increased risk of EDS.

## 1. Introduction

Emergency medical centers never close their doors, and emergency physicians are always ready to meet the patients, day or night, whether they are emergent or not. Emergency physicians are night-shift workers. Many studies have reported the risks related to night-shift work, such as hypertension, coronary artery disease, metabolic disease, substance abuse, and depression [1,2,3,4]. However, one significant issue of night-shift work is sleep disruption, such as sleep restriction, sleep deprivation, and extended wakefulness [4]. Sleep disruption has been reported to severely affect cognitive and psychomotor function [5]. Sleep disruption lowers attention and working memory; thus, acute sleep deprivation may make the subject more susceptible to errors [6].

However, excessive daytime sleepiness (EDS) of medical personnel following circadian rhythm disturbance is another critical issue since it can threaten patients’ and medical personnel’s safety. In a study of Japanese physicians having on-call/overnight working, the prevalence of EDS was 3.5% which is lower than the prevalence of multiethnic Asian adults (9.2%); however, 19.0% of physicians had experienced a medical incident within the previous month [7,8]. In a study of Chinese nurses, the prevalence of EDS (ESS ≥ 14) was 16.1%; 45.9% of nurses had experienced adverse events within the past 12 months [9]. EDS was associated with an increased risk of motor vehicle accidents [10,11]. A study of Saudi Arabia adults reported about 10% of respondents had sleep-related motor vehicle accidents or near-miss in the preceding year [12]. These risks are not an exception for emergency physicians.

Factors associated with EDS differed from study populations. A study on EDS in Australian workers found that factors associated with EDS were around the age of 50, higher body mass index, poorer diet, and poorer mental health [13]. In Brazil’s study, family income was associated with EDS [14]. However, few studies have reported factors associated with EDS in emergency physicians.

In the past 25 years, board-certified emergency physicians in Korea have grown to more than two thousand. However, there are no studies on EDS prevalence and associated factors among emergency physicians. Moreover, few studies have reported the prevalence of EDS in a single emergency physician group. The purpose of this study was to report the prevalence of EDS and identify the factors associated with EDS among emergency physicians in South Korea.

## 2. Materials and Methods

### 2.1. Study Population

We retrospectively analyzed the Korean Emergency Physicians Survey (KEPS) data. The Korean Society of Emergency Medicine (KSEM) has investigated its members’ workload, working environment, lifestyles, and health status every five years since 2010 [15]. The last survey was distributed via email and mobile phone messages to all emergency physicians registered as KSEM members from 15 January to 26 February 2021. A total of 2138 emergency physicians were registered in KSEM during the survey period. We used a sample size calculator, and the calculated sample size for the survey was 326, with a 95% confidence level and 5% margins of error [16].

### 2.2. Study Variables and Measurements

EDS was evaluated using the Epworth sleepiness scale (ESS), a widely used tool to assess recent daytime sleepiness. The ESS is a self-administered questionnaire with eight questions. Participants were asked to rate their likelihood of falling asleep on a 4-point scale (0–3) while engaging in eight different activities. The ESS scores range from 0 to 24. A higher ESS score indicates a higher average sleep propensity in daily life or daytime sleepiness [17]. The Korean version of the ESS was used in the survey, previously validated in the Korean language [18]. An ESS score of 11 or more indicated the presence of EDS.

The following variables, presumed to be associated with EDS, were collected from the survey data and compared between the groups with EDS and without EDS [13,19]. Demographic variables (age, gender, and marital status), lifestyle variables (alcohol consumption, smoking, and amount of exercise per week), and dietary habits (regular breakfast and regular night snacks) were assessed with a 5-point Likert scale, such that 5 indicated “strongly agree” and 1 indicated “strongly disagree.” To examine workloads and working environment, the annual number of patients visiting the emergency department (ED volume), monthly working days, monthly night-shift days, and monthly working hours were surveyed by self-reporting. Subjective awareness of ED safety and a guarantee of mealtime (mealtime guaranteed) were evaluated. Satisfaction with the shift schedule (schedule satisfaction), income (income satisfaction), and job (job satisfaction) were also used as working environment indicators. The questions were rated on a 5-point Likert scale, such that 5 indicated “very satisfied” and 1 indicated “very unsatisfied.” To examine sleep- and health-related variables, average sleep hours for off-duty days and self-reported sleep quality were analyzed. The question was, “How do you rate your sleep quality?” and were rated on a 5-point Likert scale from very satisfied to very unsatisfied. In addition, self-perceived health status (health perspective) was investigated on a 5-point scale. The KEPS included the adult APGAR (Access, Priorities, Growth, Assistance, and Responsibility) and Patient Health care Questionnaire-9 (PHQ-9) for screening mental health and depression. The adult APGAR (consisting of five items including components of Access, Priorities, Growth, Assistance, and Responsibility, a total of 10 points) is a brief, self-scoring instrument designed to assist physicians in assessing and monitoring their wellness status [20]. An APGAR of 5 or less is considered in a state of stress. The PHQ-9 is a self-administered questionnaire with nine questions to screen for depression and its severity. Each item is scored on a scale of 0–3 points for 27 points. The KEPS used the Korean version of the PHQ-9, previously validated in the Korean population [21]. A score of 11 or greater indicated the presence of depression.

### 2.3. Study Outcomes

The primary outcome of the study was the prevalence of EDS among emergency physicians. The secondary outcome was the factors associated with EDS.

### 2.4. Statistical Analysis

Missing data unavoidable in the survey study were addressed using Little’s MCAR test [22]. The ratio of missing data for each variable ranged from 0.3% (sleep quality) to 7.5% (job satisfaction), and the total number of missing values was 16%. Therefore, Little’s MCAR test was performed, and it was not significant (χ^2^ = 428.682, df = 355, *p* < 0.01). This indicates that these data were not missing completely at random and that multiple imputations are appropriate for the dataset. Multiple imputations were conducted for missing values with MAR. Five datasets were calculated and analyzed with a pooled dataset. Descriptive statistics were performed for demographic and EDS-related variables. Independent t-tests and chi-squared tests were performed to determine differences between groups with and without EDS. A multivariable logistic regression analysis using the “Enter” method (all variables in a univariate model are entered in a single step) was performed to calculate odds ratios (ORs) at 95% confidence intervals (CIs) and investigate the association between EDS and other variables. The mediation effect between the associated factors was analyzed by Baron and Kenny’s three-stage mediation analysis [23]. A two-sided *p* value of <0.05 was considered statistically significant. All statistical analyses were performed using SPSS 26 (IBM, Armonk, NY, USA).

## 3. Results

**Figure 1:**
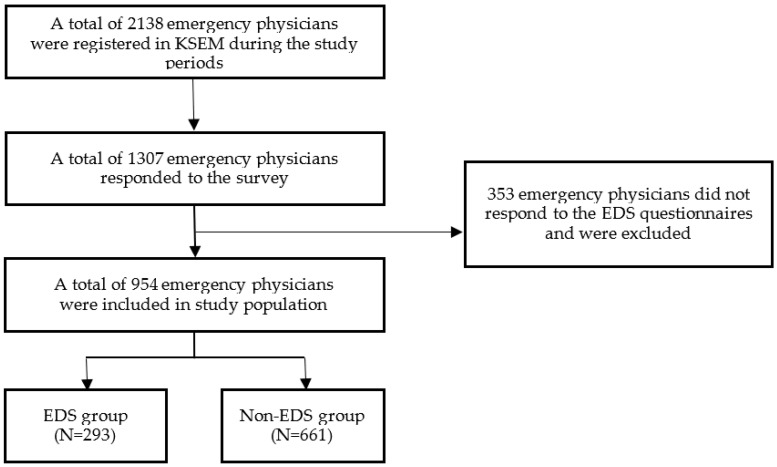
Flow chart of the study population. EDS = excessive daytime sleepiness.

A total of 1307 emergency physicians responded to the survey, and the response rate was 61.3% (1307/2138). However, 353 did not respond to the EDS questionnaire and were excluded. Finally, 954 participants were included in the study population. Among them, 293 participants were classified as the EDS group, and 661 were classified as the non-EDS group (Figure 1).

### 3.1. Characteristics of Participants

The mean age was 42.5 years, and 88.7% were men. The participants’ demographic, lifestyle, workload, working environment, and sleep-related variables are presented in Table 1.

### 3.2. Prevalence of EDS

**Figure 2:**
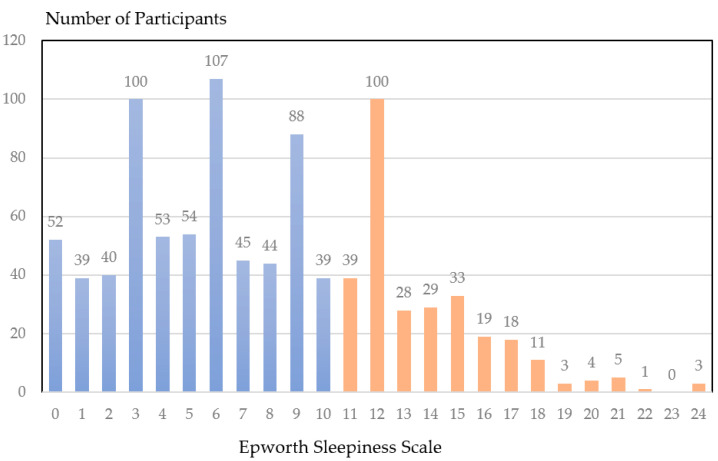
Distribution of the Epworth sleepiness scale among the emergency physicians. The *x*-axis is the Epworth sleepiness scale, and the *y*-axis is the number of participants. An Epworth sleepiness scale of 11 or more colored orange indicated the presence of excessive daytime sleepiness.

The prevalence of EDS was 30.7% (95% CI, 27.8–33.6%) (Table 1). The ESS score distribution of participants is shown in Figure 2. The results revealed that EDS was more prevalent in participants who smoke, have regular night snacks, work in more ED volumes, have more monthly night-shift days and less guaranteed mealtime, are less satisfied with schedule and income, and have poor sleep quality and perceive themselves as unhealthy. They showed a significant difference in APGAR and PHQ-9 scores compared with the non-EDS group.

### 3.3. Factors Associated with EDS

Univariable and multivariable logistic regression analyses revealed factors associated with EDS (Table 2). In the univariable analysis, smoking, regular night snack habits, monthly night-shift days, poor sleep quality, and health perspectives were associated with an increased risk for EDS. A stress condition with an APGAR score of 5 or less and depression with a PHQ-9 score of 10 or less were significantly increased risks for EDS. In contrast, guaranteed mealtime, schedule, income satisfaction, and a positive perception of health were associated with a decreased risk of EDS.

However, in the multivariable regression model, monthly night-shift days and depression were significantly associated with an increased risk of EDS, and fair health status was associated with a decreased risk of EDS. It was possible to consider that night-shift days acted as a mediator of depression rather than an independently associated factor of EDS. Thus, the mediation effect of night-shift days on depression was analyzed. Baron and Kenny’s three-stage mediation analysis was applied. In step 1, the correlation between night-shift days and depression was insignificant in both non-respondent (r = 0.01) and the respondent group (r = 0.09). There was no mediating effect on depression or a possible mediating effect on depression. Monthly night-shift days and depression were independently associated factors for EDS without mediating effects in this study.

## 4. Discussion

To the best of our knowledge, this study is the first to report EDS prevalence and associated factors among emergency physicians in South Korea. It was conducted nationwide, and the survey response rate was sufficiently high (approximately 61%). We, therefore, consider that the participants were fairly representative of emergency physicians throughout South Korea. The prevalence of EDS was 30.7%, and factors that increased the risk of EDS were monthly night-shift days, depression (PHQ-9 ≥ 11), and a decreased risk of EDS was fair sleep quality.

The prevalence of EDS among the general population in Korea has been reported to be 12.2% [24]. The prevalence of EDS among emergency physicians in this study is more than twice that of the general population in Korea. The prevalence of EDS among physicians ranges widely from study to study. However, most studies report that the EDS prevalence among physicians is higher than that of the general population. A study of EDS on academic physicians reported that 28.6% of physicians felt sleepy during the daytime (ESS score > 10) [25]. Another study of EDS on attending physicians reported that 15.9% of physicians were found to be sleepy (ESS score > 10) [26]. The EDS prevalence (ESS > 10) of the emergency physician in Saudi Arabia was 41.2% [27]. According to the Korean Emergency Medicine Resident Survey 2020, the prevalence of EDS among emergency medicine residents was 32.4% [28]. The prevalence of EDS among emergency physicians is certainly higher than that of the general population and physicians of other specialties.

In this study, the significant factors associated with developing EDS were depression and monthly night-shift days. A study of British workers showed that most can synchronize their circadian systems to night shifts within a week [29]. However, it can take weeks for workers to re-entrain to day shifts [30,31,32]. This means that as the number of monthly night shifts increases, there will not be enough time to restore their circadian rhythm, which will eventually lead to EDS. Frequent night shifts and consequent sleep deprivation increase the risk for major depression [33]. Depression as a risk factor for EDS has also been reported in other studies [34,35]. However, this study found that monthly night-shift days and depression were independently associated factors for EDS without mediating effects. The causal relationship between EDS and night-shift days is intuitively clear. However, whether depression was the cause or result of EDS was not established. Further research is needed on whether other factors cause depression or EDS causes depression.

How many night shifts in a month would be appropriate for emergency physicians to work without EDS? The tolerable number of night shifts may vary depending on the workload and working environments of each emergency medical center and emergency physician. This study showed a small but significant difference in night-shift days between the EDS and the non-EDS group (6.2 ± 2.2 versus 5.7 ± 2.5, *p* = 0.007). In Korea’s current emergency medical service system, it is estimated that night shifts more than six times a month are not tolerable for emergency physicians. However, in a study of EDS among emergency medicine residents in Korea, the monthly night-shift days did not significantly differ between the EDS and non-EDS groups. They were not associated factors [26]. The study explained this finding by noting that the working hours of residents were limited to less than 80 h per week, and rest time the next day of the night shift was guaranteed by a policy for the improvement of training conditions and status of medical residents, enacted and enforced since 2016 [36,37]. As trainees, emergency medicine residents usually work with supervisors and their peers. However, emergency physicians in local emergency medical centers of non-training hospitals usually work alone. The difference between an emergency physician and an emergency medicine resident is considered the difference in the working environment.

Although this is the first nationwide report on EDS among emergency physicians in Korea, it has several limitations. First, there is still a possibility of nonrespondent bias (selection bias due to nonrespondents) inherent to survey research. Second, this study investigated self-perceived EDS, and no objective assessment of sleep problems was conducted. Physicians in the EDS group did not differ in sleep hours but in sleep quality. Thus, EDS appears more susceptible to quality than quantity of sleep [38]. However, we did not investigate which sleep problems, including sleep initiation, sleep maintenance, and sleep disorders, such as sleep apnea, appeared in participants. In future studies, objectively measuring sleep quality using polysomnography will be necessary.

In conclusion, according to this study, the prevalence of EDS among emergency physicians in Korea is about three times higher than that of the general population, and almost one in three emergency physicians suffers daytime sleepiness. The number of monthly night-shift days and depression were associated with an increased risk of EDS. The health status of the medical staff is closely related to not only the patient’s clinical outcome but also the patient’s safety. We suggest that follow-up qualitative studies on the cause of depression and prospective longitudinal studies on the workload and working environment, including monthly night-shift days, are necessary.

## Figures and Tables

**Table 1:** Characteristics of participants.

	Participants	Total Group (N = 954)	EDS Group (N = 293)	Non-EDS Group (N = 661)	*p*-Value
Demographic variables					
Age	954	42.5 ± 6.1	42.1 ± 5.7	42.7 ± 6.3	0.175
Gender	954				0.112
Men		846 (88.7)	267 (91.1)	579 (87.6)	
Woman		108 (11.3)	26 (8.9)	82 (12.4)	
Marital status	945				0.632
Married		832 (88.0)	254 (87.3)	578 (88.4)	
Others		113 (12.0)	37 (12.7)	76 (11.6)	
Lifestyle variables					
Alcohol consumption	945				0.233
No		566 (59.9)	166 (57.0)	400 (61.2)	
Yes		379 (40.1)	125 (43.0)	254 (38.8)	
Smoking	948				0.034
No		702 (74.1)	203 (69.5)	499 (76.1)	
Yes		246 (25.9)	89 (30.5)	157 (23.9)	
Number of exercises per week	954	1.8 ± 1.8	1.7 ± 1.9	1.8 ± 1.8	0.367
Regular breakfast ^a^	947	2.5 ± 1.4	2.5 ± 1.3	2.5 ± 1.4	0.561
Regular night snacks ^a^	947	2.7 ± 1.2	2.9 ± 1.2	2.6 ± 1.3	0.004
Workloads and working environment variables					
ED volume ^c^	884	34,375.6 ± 20,317.8	36,280.8 ± 20,831.2	33,393.9 ± 21,975.8	0.042
Monthly working days	954	11.7 ± 4.2	11.6 ± 3.8	11.7 ± 4.3	0.918
Monthly night-shift days	954	5.9 ± 2.4	6.2 ± 2.2	5.7 ± 2.5	0.007
Monthly working hours	954	152.8 ± 51.0	151.1 ± 57.9	153.6 ± 47.6	0.480
ED safety ^a^	903	2.9 ± 1.0	2.8 ± 1.0	3.0 ± 1.1	0.054
Mealtime guaranteed ^a^	950	2.5 ± 1.2	2.4 ± 1.1	2.6 ± 1.2	0.013
Schedule satisfaction ^b^	949	3.4 ± 0.9	3.3 ± 0.9	3.5 ± 0.9	<0.001
Income satisfaction ^b^	954	2.8 ± 1.0	2.6 ± 1.0	2.8 ± 1.0	0.008
Job satisfaction ^b^	882	3.2 ± 0.9	3.2 ± 0.9	3.3 ± 1.0	0.235
Sleep and health-related variables					
Sleep hours	954	7.3 ± 1.4	7.3 ± 1.4	7.4 ± 1.4	0.285
Sleep quality	951				<0.001
Not very worried		80 (8.4)	15 (5.1)	65 (9.9)	
Not worried		193 (20.3)	37 (12.7)	156 (23.7)	
Fair		232 (24.4)	64 (21.9)	168 (25.5)	
Worried		293 (30.8)	109 (37.3)	184 (27.9)	
Very worried		153 (16.1)	67 (22.9)	86 (13.1)	
Health perspective	949				<0.001
Very unhealthy		26 (2.7)	13 (4.5)	13 (2.0)	
Unhealthy		178 (18.8)	81 (27.8)	97 (14.7)	
Fair		423 (44.6)	121 (41.6)	302 (45.9)	
Healthy		280 (29.5)	68 (23.4)	212 (32.3)	
Very healthy		42 (4.4)	8 (2.7)	34 (5.2)	
Wellness	907				<0.001
APGAR score > 5		311 (34.3)	67 (23.8)	244 (39.0)	
APGAR score ≤ 5		596 (65.7)	215 (76.2)	381 (61.0)	
Depression	908				<0.001
PHQ-9 < 11		650 (71.6)	147 (51.9)	503 (80.5)	
PHQ-9 ≥ 11		258 (28.4)	136 (48.1)	122 (19.5)	

Variables are presented as the mean ± standard deviation and number (%). The survey questions on regular breakfast ^a^, regular night snack ^a^, ED safety ^a^ and mealtime guaranteed ^a^ were developed on a 5-point Likert scale; 5 points were strongly agreed upon, and 1 point was strongly disagreed. The survey questions on schedule satisfaction ^b^, income satisfaction ^b^, and job satisfaction ^b^ were developed on a 5-point Likert scale; 5 points were very satisfied, and 1 point was very unsatisfied. ED volume ^c^ means the annual number of patients visiting the emergency medical center. EDS = excessive daytime sleepiness, ED = emergency department, APGAR score = access priorities growth assistance responsibility score, PHQ-9 = patient health care questionnaire-9, ESS = Epworth sleepiness scale.

**Table 2:** Factors associated with EDS.

	Univariable Logistic Regression		Multivariable Logistic Regression	
	OR	95% CI	OR	95% CI
Demographic variables				
Age	0.984	0.962–1.007	0.987	0.957–1.017
Gender				
Male	1.000		1.000	
Female	0.688	0.432–1.094	1.714	0.996–2.949
Marital status				
Married	1.000		1.000	
Others	1.065	0.723–1.099	1.062	0.650–1.736
Lifestyle variables				
Alcohol consumption				
No	1.000		1.000	
Yes	1.192	0.900–1.579	0.909	0.656–1.259
Smoking				
No	1.000		1.000	
Yes	1.389	1.022–1.888	0.786	0.548–1.125
Number of exercises per week	0.966	0.895–1.042	1.050	0.962–1.147
Regular breakfast ^a^	0.970	0.876–1.074	1.807	0.964–1.226
Regular night snack ^a^	1.177	1.052–1.317	1.080	0.952–1.224
Workloads and working environment variables				
ED volume ^c^	1.000	1.000–1.000	1.000	1.000–1.000
Monthly working days	0.998	0.966–1.032	0.995	0.953–1.040
Monthly night-shift days	1.084	1.022–1.149	1.106	1.028–1.191
Monthly working hours	0.999	0.996–1.002	0.998	0.994–1.001
ED safety ^a^	0.870	0.756–1.002	0.891	0.749–1.060
Mealtime guaranteed ^a^	0.864	0.769–0.970	0.965	0.844–1.105
Schedule satisfaction ^b^	0.760	0.651–0.888	0.959	0.774–1.188
Income satisfaction ^b^	0.827	0.718–0.953	0.904	0.743–1.100
Job satisfaction ^b^	0.911	0.780–1.063	1.209	0.989–1.478
Sleep and health-related variables				
Sleep hours	0.947	0.856–1.047	0.988	0.882–1.106
Sleep quality				
Not very worried	1.000		1.000	
Not worried	1.035	0.531–2.019	0.634	0.305–1.319
Fair	1.648	0.874–3.106	0.560	0.318–0.985
Worried	2.569	1.391–4.745	0.796	0.486–1.303
Very worried	3.366	1.760–6.436	0.996	0.638–1.556
Health perspective				
Very unhealthy	1.000		1.000	
Unhealthy	0.842	0.369–1.921	1.970	0.565–6.870
Fair	0.410	0.184–0.911	1.666	0.661–4.200
Healthy	0.328	0.145–0.740	1.262	0.526–3.041
Very healthy	0.236	0.080–0.700	1.416	0.590–3.398
Wellness				
APGAR score > 5	1.000		1.000	
APGAR score ≤ 5	2.029	1.484–2.776	1.395	0.953–2.043
Depression				
PHQ-9 < 11	1.000		1.000	
PHQ-9 ≥ 11	3.570	2.635–4.835	2.635	1.799–3.861

The survey questions on regular breakfast ^a^, regular night snack ^a^, ED safety ^a^, and mealtime guaranteed ^a^ were developed on a 5-point Likert scale; 5 points were strongly agreed upon, and 1 point was strongly disagreed. The survey questions on schedule satisfaction ^b^, income satisfaction ^b^, and job satisfaction ^b^ were developed on a 5-point Likert scale; 5 points were very satisfied, and 1 point was very unsatisfied. ED volume ^c^ means the annual number of patients visiting the emergency medical center. EDS = excessive daytime sleepiness, ED = emergency department, APGAR score = access priorities growth assistance responsibility score, PHQ-9 = patient health care questionnaire-9, OR = odds ratio, CI = confidence interval.

## Data Availability

The data supporting this study’s findings are available on request from the corresponding author. The data are not publicly available because they contain information that could compromise the privacy of research participants.
